# Antioxidant and antibacterial insights into the leaves, leaf tea and medicinal roots from *Astragalus membranaceus* (Fisch.) Bge.

**DOI:** 10.1038/s41598-021-97109-6

**Published:** 2021-10-04

**Authors:** Anim Okyere Samuel, Bao-Ting Huang, Yuan Chen, Feng-Xia Guo, Dou-Dou Yang, Jian-Qin Jin

**Affiliations:** 1 College of Life Science and Technology, College of Agronomy, Gansu Provincial Key Lab of Aridland Crop Science, Gansu Key Lab of Crop Genetic Improvement & Germplasm Enhancement, Gansu Provincial Key Lab of Good Agricultural Production for Traditional Chinese Medicines, Gansu Provincial Engineering Research Centre for Medical Plant Cultivation and Breeding, Gansu Agricultural University, Lanzhou, 730070 China; 2 CSIR-Oil Palm Research Institute, P.O BOX 74, Kusi, Ghana

**Keywords:** Ecology, Health care

## Abstract

Used as traditional Chinese medicine, *Astragalus membranaceus* (Fisch.) Bge. (*A. membranaceus*) roots are also used as tonic food material in a wide range of applications, while the leaves are left in the field, unused. Therefore, comprehensively exploring and utilizing the leaves will inevitably reduce the associated resource waste and environment pollution. In this study, the plant leaves were processed into tea using green tea processing technology. Bioactive components, antioxidant and antibacterial activities of the Leaf Tea (LT) and Dry Leaves (DL) were studied, and compared to that of the Dry Roots (DR). The results showed that the polysaccharides content (POL) in the DR (20.44%) was twice as high as the DL (10.18%) and LT (8.68%). However, the DL contained 36.85% more water-soluble extracts (WSE), 35.09% more ethanol-soluble extracts (ESE), 409.63% more total flavonoid content (TFC), 221.01% more total phenolic content (TPC) and 94.34% more proteins, and the LT contained 26.21% more WSE, 40.64% more ESE, 326.93% more TFC, 191.90% more TPC and 37.71% more proteins. The total amino acid (AA) content in the DR was 8.89%, while in that of the DL and LT were 24.18% and 28.96% respectively, nearly 3-times higher than that of the DR. The antioxidant activity of DR was much lower than those of DL and LT, both of which had antioxidant activity closer to that of Vitamin C (V_C_) and the antioxidant activities were even stronger when the optimal concentration was reached. Except for *Aspergillus niger* and *Staphylococcus aureus,* the DL and DR exhibited inhibition activities to *Salmonella*, *Bacillus subtilis, Escherichia coli* and *yeast*, while the LT had antimicrobial activities against all the strains except for *A. niger*. In summary, compared with the most commonly used DR, the DL and LT from *A. membranaceus* contained higher bioactive components, and stronger antioxidant and antimicrobial activities. Producing leaf tea may be an appropriate way to economically and reasonably utilize the plant leaves which are by-products.

## Introduction

Astragali Radix (Huangqi) is the dry roots of the leguminous *Astragalus membranaceus* (Fisch.) Bge. var. *mongholicus* (Bge.) Hsiao (*A. membranaceus* var. *mongholicus*) or *Astragalus membranaceus* (Fisch.) Bge. (*A. membranaceus*)^1^. It has been used as traditional medicine and tonic food material in China, Japan, Korea, and some Southeast Asia countries for more than 2000 years^2,3^, It can reinforce body immunity and has curative effect on (diuresis and edema, stagnation and obstruction as well as detoxification and purulence^4,5^). In addition, Astragali Radix has tonifying middle and Qi, and is used to treat deficiency, vitalise your energy and relief fatigue, eating less and loose stools^1,6^, which can improve anti-viral activity, protect liver and stomach^7–9^, inhibit cancer^10^, exert anti-aging and improve exercise performance^5^. Based on the above functions, Astragali Radix was recommended by the National Administration of Traditional Chinese Medicine^11^ as a Chinese traditional herbal medicine in the prevention of infection associated with the novel coronavirus COVID-19.

In recent years, there have been many reports on the chemical constituents and pharmacological active ingredients, including polysaccharides^12^, saponins^13^, amino acids, trace elements and flavonoids^14^ of the dry roots of *A. membranaceus*^3^. Similar to the roots, *A. membranaceus* leaves also possess medicinal or health promoting benefits. However, evidence of the bioactive components, antioxidant and antibacterial activities of the leaves of this medicinal plant is very limited. Additionally, little comparative studies exist between the leaves and the roots. Astragali Radix is an integral component in many pharmaceutical, beverage, and cosmetics industries^2,15^. During its cultivation, some the shoots and leaves are cut off to promote underground growth and bioactive compound biosynthesis. The removed leaves from *A. membranaceus* are underutilized in most producing areas^16^. Based on ancient record and modern science studies, *A. membranaceus* leaves can be used as a food source^17–19^. To promote *A. membranaceus* leaves as a nourishing healthy food resource, a comprehensive scientific study of the leaf processing procedures, the quantity and types of active ingredients they contain must be undertaken.

Therefore, the purpose of this study was to detect and comparatively analyze the bioactive constituents of *A. membranaceus* leaves, leaf tea and roots, and to ascertain their antioxidant and antibacterial activities in vitro.

## Materials and methods

### Plant materials and product processing

The *A. membranaceus* plants were cultivated by local farmers in Huining, Gansu province, P.R. China (35° 40′ N, 105° 21′ E), samples collection did not require permission. The fresh leaves of *A. membranaceus* were sampled in June and transported to laboratory for processing while the roots were harvested on Oct. 20, 2019. The plants were cultivated in summer 2017 and were harvested for the shoots in June 2019. The shoots were cut 20 cm above the ground level.

The leaf tea (LT) was processed according to the standard green tea processing procedure^20^. The carefully selected healthy-fresh leaves were placed on a cloth for 2 h to dissipate some moisture, blanched for 6 min at 200 °C, rolled by hand and oven dried to constant weight at 60 °C for 3 days to make the dry leaf product (DL). The healthy fresh roots were oven dried to constant weight at 60 °C to make the dry root product (DR).

**Figure 1:**
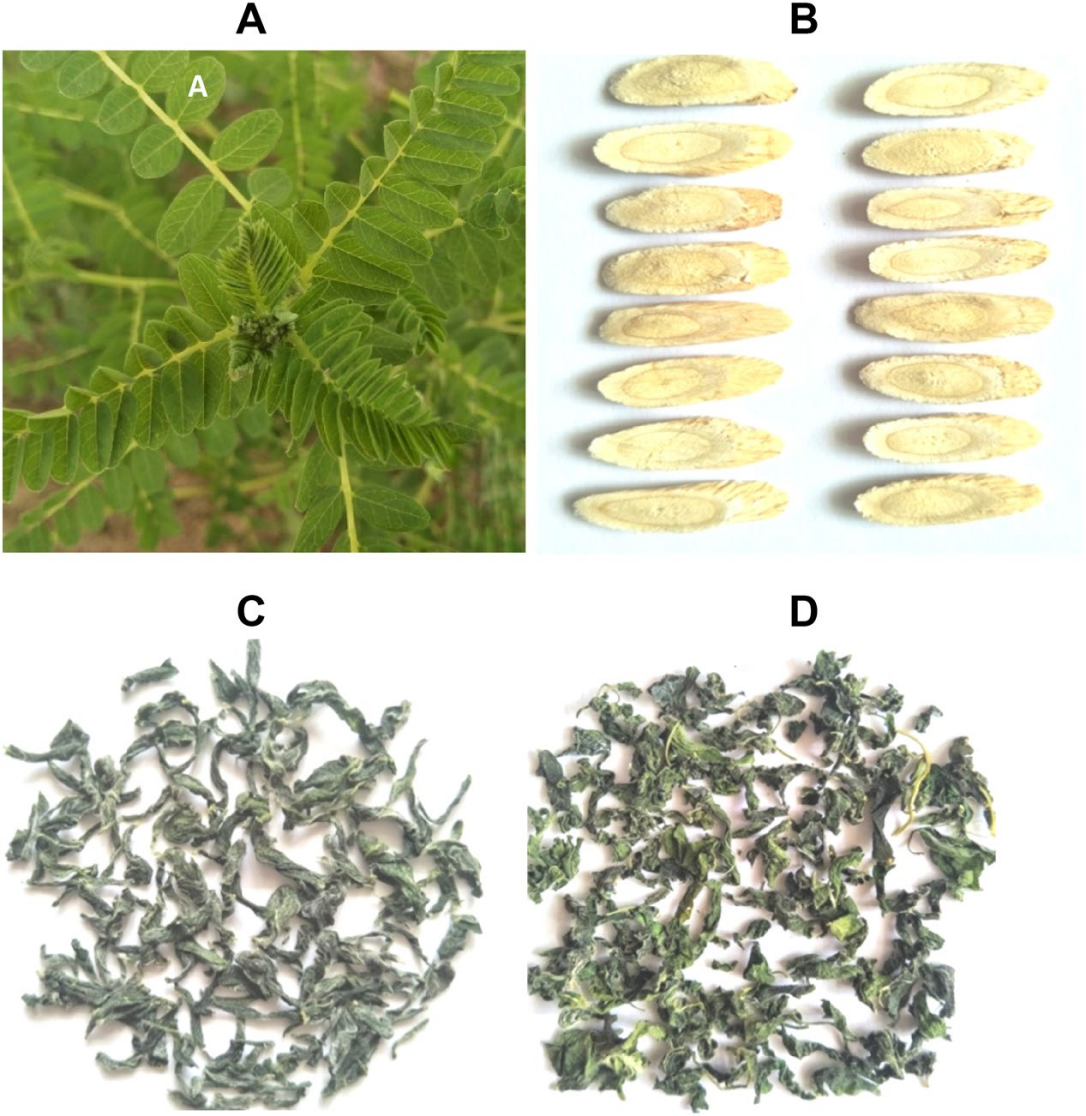
Morphology of plant leaves growing in a field (**A**), dry roots (**B**), leaf tea (**C**) and dry leaves (**D**) processed from *A. membranaceus*.

All the above products, LT, DL and DR, Fig. 1, were crushed separately, sifted through 80 mm meshes, packaged in self-sealing bags, and stored at − 80 °C for subsequent analysis.

### Chemicals and reagents

The chemicals H_3_PO_4_, K_2_S_2_O_8_, K_4_Fe (CN)_6_·3H_2_O, NaNO_2_, NaOH, Na_2_CO_3_, CuSO_4_, K_2_SO_4_, H_3_BO_3_, AlCl_3_, NaH_2_PO_4_, Na_2_HPO_4_, H_2_SO_4_, HCl, vitamin C (V_C_), methanol, ethanol and phenol were purchased from Shanghai Hushi Chemical Ltd (Shanghai, China). Beef peptone was purchased from AOBOX (Beijing, China). Gallic acid and rutin were purchased from Solarbio (Beijing, China). Folin & Ciocalteu's phenol reagent were purchased from Sigma (America). DPPH, agar powder and 2,2′-azino-bis(3-ethylbenzothiazoline-6-sulfonic acid) were purchased from Tokyo Chemical Industry Co., Ltd (Tokyo, Japan).

### Bioactive composition determination

Contents of polysaccharides, protein, moisture and ash of the samples were determined according to the Association of Official Analytical Chemists (AOAC) methods^20,21^.

The total phenol content (TPC) was analyzed using the Folin-Ciocalteu (F–C) reagent method. A sample of the 0.10 g powder of each product received 3 mL of 70% methanol at 70 °C and extracted three times at 10 min interval using the sonic wave degradation method. The samples were centrifuged for 10 min at 3500 r/min, and the supernatant of three times centrifugation was transferred to a 10 mL volumetric bottle. The absorbance of the reaction mixture was measured at 760 nm using a spectrophotometer (Shimadzu UV-2450, Japan)^20,22^. Absorbance values were converted to contents using a standard curve expressed by gallic acid (*y* = 0.047 + 0.478*x; r*^2^ = 0.999).

Total flavonoid content (TFC) detection was based on the AlCl_3_ colorimetric method^20,23^. A 0.10 g sample was mixed with 3 mL 95% ethanol at 50 °C. The supernatant was extracted three times by ultrasound for 30 min each time, and centrifuged for 10 min at 4000 r/min. The supernatant was transferred to a 10 mL volumetric bottle. The absorbance of the sample was measured at 510 nm. The absorbance was converted to content using a standard curve expressed by rutin (*y* = 0.005 + 0.294*x; r*^2^ = 0.999). The ethanol- and water-soluble extracts were measured according to the Chinese Pharmacopoeia method^1^. A 2.0 g sample was put in a 100 mL Erlenmeyer flask containing 50 mL water (for the water-soluble extract) or 45% ethanol (for the ethanol-soluble extract). The plug was closed and connected to a reflux condenser, heated for 1 h to slightly boil and filtered. The residue of the 25 mL sample was oven dried to constant weight at 60 °C.

As referenced in the national standard GB 5009.124-2016^24^, amino acid contents of the DL, LT and DR, (Fig. 1), from *A. membranaceus* were determined by automatic amino acid analyzer (Hitachi L-8900, Japan).

### Antioxidant activity determination

For the antioxidant activity analysis, samples were prepared according to the following method: A 1.0 g sample and 50 mL pure water (water extract antioxidant activity analysis) or 70% ethanol (ethanol extract antioxidant activity analysis) were put into a 100 mL Erlenmeyer flask, the plug closed and heated to slight boiling for 1 h. The extract was transferred to a 50 mL volumetric bottle, using V_C_ as the positive control.

### 2,2-Azino-bis-(3-ethyl-benzothiazoline-6-sulfonic acid) radical scavenging activity

A 2,2-azino-bis-(3-ethyl-benzothiazoline-6-sulfonic acid) (ABTS) radical solutions were prepared as follows: 4.0 mL 7.0 mM ABTS and 2.0 mL 7.0 mM potassium per sulfate were mixed together. The mixture was kept at room temperature in dark for 12 h, while ABTS cation formed. The sample was then diluted before analysing with ethanol to give an absorbance of 0.700 ± 0.02 at 734 nm. The reaction mixture was developed by mixing a 10 μl product sample or control (70% methanol) with a 1.0 mL ABTS radical solution. Samples and blanks were placed in vortexes for 6 min, and measured at 734 nm. A 70% methanol was used as the blank^20^. The ABTS radical scavenging activity expressed as a percent was calculated as follows:

$${\text{ABTS}}\;{\text{radical}}\;{\text{inhibition }}\left( \% \right) = [1 - {\text{sample A}}_{{734{\text{nm}}}} /{\text{control A}}_{{734{\text{nm}}}} ] \times 100$$

### Inhibition of 2,2-diphenyl-1-picrylhy-drazyl (DPPH^⋅^) radicals

2,2-diphenyl-1-picrylhy-drazyl (DPPH^⋅^) was dissolved in methanol to 0.2 mM. 2 mL of the solution were mixed with 2 mL of the diluted sample at 25 °C for 30 min. At the end of the reaction, the absorbance was read at 510 nm^20,25^. Inhibiting percentages of the DPPH^⋅^ radical were calculated with the following formula.

$${\text{DPPH}}^{ \cdot } \;{\text{radical inhibition rate }}\left( \% \right) \, = \, [{\text{Ao}} - \left( {{\text{Ax}} - {\text{Axo}}} \right)]/{\text{Ao}} \times {1}00\%$$

where: Ao is absorbance of the mixed reaction solution with 2 mL DPPH^⋅^ and 2 mL water; Ax is absorbance of the final DPPH^⋅^ inhibition reaction solution with a 2 mL sample and 2 mL DPPH^⋅^; Axo is absorbance of the agent used in the test with no DPPH^⋅^ but a 2 mL sample and 2 mL pure water.

### Ferric ion reducing antioxidant power (FRAP)

Reducing power of samples were measured using FRAP assay^20,26^. In a nutshell, a FRAP reagent was freshly prepared by mixing an acetate buffer (0.3 M, pH 3.6). 1.0 mL of FRAP reagent and 0.05 mL of properly diluted sample solution were added and mixed well. After reaction for 30 min at 37 °C, the absorbance was read at 593 nm. FRAP was expressed by V_C_ (*y* = − 0.013 + 0.006*x, r*^2^ = 0.998).

### Antibacterial activities

#### Microbial strains sources and the tested product sample preparation

Six microbial strains including Salmonella, *Bacillus subtilis (B. subtilis), Staphylococcus aureus (S. aureus), Escherichia coli (E. coli), yeast, Aspergillus niger (A. niger)* were obtained from the Northwest Institute of Ecology and Environmental Resources, Chinese Academy of Sciences.

Samples were prepared as follows: a 1.0 g sample and 50 mL distilled water were put into a 100 mL conical bottle. The bottle was sealed and heated to boil for 1 h and the extract was transferred to a 50 mL volumetric bottle.

#### Microbial activation and antibacterial activity test

Salmonella, *B. subtilis*, *S. aureus*, *E. coli* were cultured on beef-protein medium for 24 h at 37 °C. *A. niger* and *yeast* were cultured on Potato Dextrose Agar (PDA) for 48 h at 28 °C. The strain density was 1.6–2.5 × 10^9^ cfu/mL. 100 μl strains were incubated in each plate.

Sterilized filter paper discs with 6 mm diameter were dipped in the sample extract and irradiated for 40 min under UV light. Then the soaked discs were placed in a *microbial* strain plate. The inhibition diameter was measured with a vernier caliper and the MIN and MAX effects were tested^27^.

### Statistical analysis

Significant differences among the treatments were analyzed by Duncan multiple comparison based on one-way ANOVA. The Regression analysis with SPSS 20.0 software, indicated that the inhibition of water-soluble extracts (WSE) and ethanol-soluble extracts (ESE) to free radical ABTS and DPPH^⋅^ were performed according to method developed in previous research^20^.

## Results and discussion

### Bioactive composition analysis

The main bioactive components in the three products are listed in Table 1. The main chemical constitutes of DL and LT were quite similar; although significant differences were noted in indicators such as protein (DL > LT, difference = 8.22, *P* < 0.01), ESE (LT > DL, difference = 1.79, *P* < 0.01) and WSE (DL > LT, difference = 4.07, *P* < 0.05). Significant differences were not found in the TPC, TFC and POL contents (*P* > 0.05) of the samples. Among the bioactive constitutes, only POL contents in LT and DL were significantly lower (*P* < 0.01) while the other components in LT and DL were all significantly higher than those in DR (*P* < 0.01). Two major anti-inflammatory active fractions that may enhance wound healing were isolated from dry roots of *A. membranaceus*^28^. The extract from *A. membranaceus* var *mongholicus* roots has shown significant anti-viral and immune stimulate activities, from which a new saponin and iso-astragaloside (I) were separated^3^ Leaf products exhibited substantial total flavonoid with contents in the order DL (9.68%) > LT (8.11%) > DR (1.90%). Compared with DL and LT, the DR exhibited significantly higher POL, increased by 50.21% (*P* < 0.01) and 57.53% (*P* < 0.01), respectively. Compared with DR, DL and LT showed other higher nutrition components, leading to increased WSE of 14.10% and 10.03%, increased ESE of 11.27% and 13.05%, increased TFC of 7.78% and 6.21%, increased TPC of 3.99% and 3.46%, and increased Protein of 13.70% and 5.48%, respectively. The abundant TPC in LT and DL detected in this study were in accordance with the results of study examining the fleshy roots of *Raphanus raphanistrum*^21^. In addition to abundant TPC, increased TFC and protein in LT and DL was also detected in this study; these results were in accordance with studies of leaf tea and dry leaf from *C. pilosula*^20^. Differing from *C. pilosula* products, the contents of WSE and ESE were reversed in LT and DR from *A. membranaceus,* resulting in a maximum amount of WSE in DL and a maximum amount of ESE in LT^20^.

**Table 1:** Bioactive components in three products from *Astragalus membranaceus* (Fisch.) Bge.

Components (%)	Different products		
	DL	LT	DR
Moisture	5.75 ± 0.42^aA^	4.58 ± 0.02^bB^	3.55 ± 0.01^cC^
Ash	7.74 ± 0.10^bB^	8.35 ± 0.05^aA^	4.37 ± 0.04^cC^
WSE	52.36 ± 0.64^aA^	48.29 ± 1.66^bA^	38.26 ± 2.26^cB^
ESE	43.37 ± 0.28^bB^	45.16 ± 0.58^aA^	32.11 ± 0.54^cC^
TPC	5.79 ± 0.03^aA^	5.26 ± 0.44^aA^	1.80 ± 0.21^bB^
POL	10.18 ± 0.79^bB^	8.68 ± 0.18^bB^	20.44 ± 0.58^aA^
Protein	28.21 ± 0.11^aA^	19.99 ± 0.29^bB^	14.52 ± 0.90^cC^
TFC	9.68 ± 0.52^aA^	8.11 ± 0.88^bA^	1.90 ± 0.10^cB^

The data are presented as the means ± SDs. DL, dry leaves; LT, leaf tea; DR, dry roots; WSE, water-soluble extract; ESE, ethanol-soluble extract; TPC, total polyphenolic content; POL, Polysaccharides; TFC, total flavonoid content. In each column, the different lowercases and uppercases mean significant difference at P < 0.05 and great significant difference at P < 0.01, respectively, based on results of ANOVA-Duncan multiple comparisons by SPSS 20.0. All the abbreviation is the same as following tables.

Amino acids are essential for human growth and development as well as reproduction and health^29^. Table 2 shows the amino acids (AA) in DL, LT and DR from *A. membranaceus*. The results showed that the 17 amino acid contents in DL and LT were all very similar leading to a non-significant difference in the total value (*P* > 0.01). Compared with DL (24.18%) and LT (28.96%), the total AA content in DR was significantly lower 8.89% (*P* < 0.01), which were nearly one third times lower than the formers. This indicates that all the three products are rich in amino acids (Table 2), because their total amino acid contents are far higher than those of famous Chinese teas including Maojian (slightly more than 0.02%), Biluochun (close to 0.03%), Longjing (slightly more than 0.03%)^30^. DR, DL and LT also presented more abundant amino acids than their respective products of dry root (5.36%), dry leaves (19.01%) and leaf tea (21.11%) from *C. pilosula*^20^.

**Table 2:** Amino acid contents in three products from *Astragalus membranaceus* (Fisch.) Bge.

Amino acids	Contents of amino acids in three products (%)		
	DL	LT	DR
Alanine	1.49	1.66	0.38
Serine	1.20	1.45	0.36
Leucine	2.10	2.35	0.49
Aspartic acid	3.51	4.52	1.78
Isoleucine	1.22	1.41	0.33
Glycine	1.27	1.50	0.36
Arginine	1.38	1.86	0.64
Histidine	0.77	0.98	0.33
Valine	1.54	1.82	0.46
Proline	1.39	1.64	1.10
Threonine	1.29	1.48	0.40
Phenylalanine	1.28	1.43	0.28
Methionine	0.07	0.08	0.02
Glutamate	2.80	3.37	1.09
Lysine	1.99	2.40	0.64
Tyrosine	0.88	1.01	0.23
The total	24.18	28.96	8.89

The average data are presented in the table. The total described the sum of contents for all the amino acids in each product. DL, the abbreviations are the same as presented in Table 1.

Low ash content is desirable for Chinese Pharmacopoeia^1^. The crude ash content showed a minimum value in medicinal DR, but in the by-products of LT and DL the values increased by 3.98% and 3.38% respectively greater than that of DR. However, this does not affect the utilization value of leaf products, because these products are consumed by soaking rather than eating leaves as a whole, while ash is insoluble in water. The total ash content is mainly constituted by the mineral element, so the mineral element contents in leaves and leaf tea were higher than those in roots^2^. Similar results were demonstrated in the products of medicinal plant *C. pilosula*^20^.

The above results show that in addition to being rich in amino acids, dry leaf and leaf tea also have higher TFC, a natural antibiotic, providing an important basis for the development and utilization of LT products in the future.

### Antioxidant activity analysis

**Figure 2:**
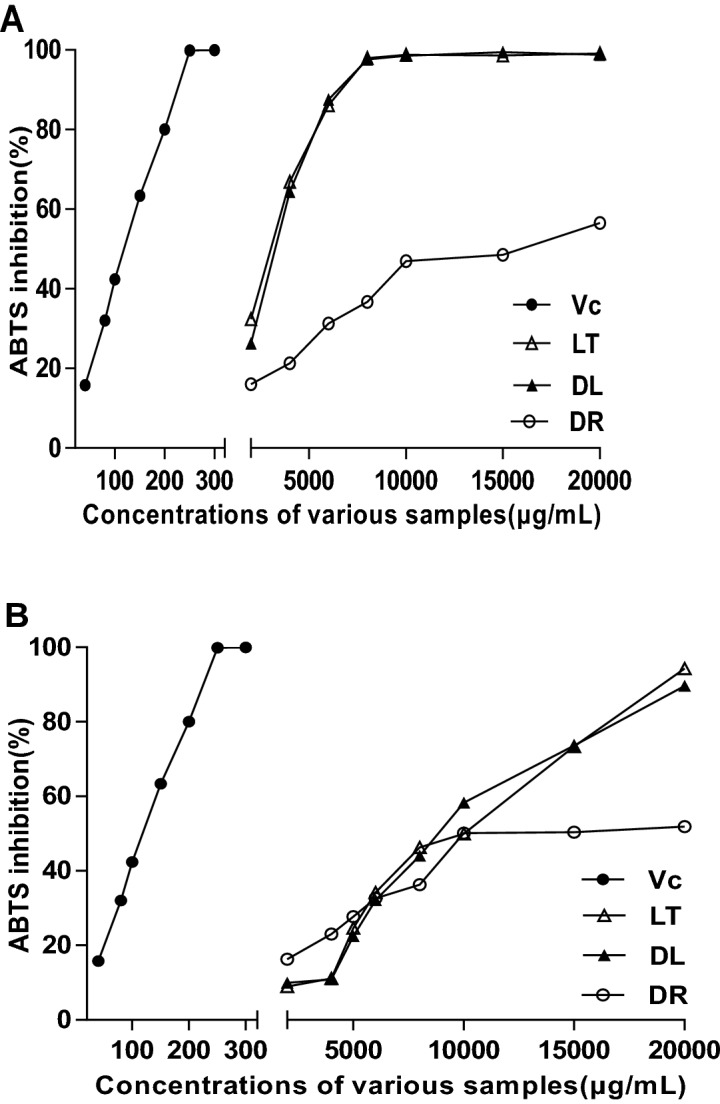
Inhibition of 2,2-azino-bis-(3-ethyl-benzothiazoline-6-sulfonic acid) (ABTS) radical for ethanol water-soluble extracts samples (**A**) and ethanol-soluble extracts samples (**B**) from DL (filled triangle), processed LT (open triangle) and medicinal DR (open circle) of *A. membranaceus*, compared with Vc (closed circle); DL, dry leaves; LT, leaf tea ; DR, dry roots; Vc, vitamin C.

The antioxidant activities of the solutions extracted from the three products were measured using ABTS, DPPH^⋅^ and FRAP, using V_C_ as a positive control. Figure 2 shows inhibitions of the samples to ABTS radicals. This could suggest that DR also presented lower scavenging activities than those in WSE and ESE from samples of DL and LT.

When 20,000 μg/mL was reached, ESE from the DR product showed a 51.89% inhibition rate to ABTS free radical, while the value of WSE was 56.52%. WSE and ESE of LT and DL had very similar scavenging ability to free radicals, and both have much stronger scavenging ability to free radicals than the respective root extracts. Once LT and DL concentration reached a certain level, the abilities noticeably reached that of V_C_. The highest inhibition rate of 99.94% in V_C_ appeared at concentration of 300 μg/mL (Fig. 2). The highest value was 99.49% at concentration of 15,000 μg/mL in DL, and 99.10% at concentration of 20,000 μg/mL in LT for WSE samples (Fig. 2A).

However, for ESE samples, the highest inhibition rates of 89.62% and 94.28% in DL and LT respectively all appeared at a concentration of 20,000 μg/mL (Fig. 2B). Therefore, we concluded that LT and DL could inhibit ABTS radical efficiently at lower concentrations than DR. Furthermore, the inhibition effect of DL and LT were quite similar, and the highest inhibitions reached as high as V_C_ at certain concentrations. The same inhibitions result also appeared in the corresponsive products of another medicinal and edible *C. pilosula*^20^.

**Figure 3:**
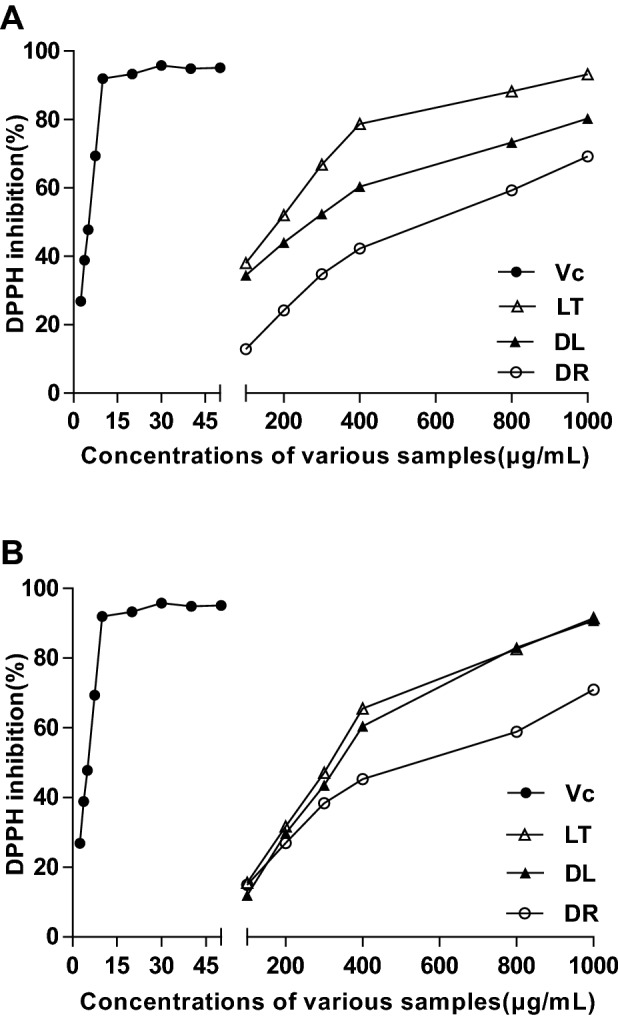
Inhibition of 2,2-diphenyl-1-picrylhy-drazyl (DPPH) radical for water-soluble extracts samples (**A**) and ethanol-soluble extracts samples (**B**) from DL (filled triangle), processed LT (open triangle) and medicinal DR (open circle) of *A. membranaceus*, compared with Vc (closed circle)); DL, dry leaves; LT, leaf tea ; DR, dry roots; Vc, vitamin C.

The DPPH^⋅^ radical inhibition ability of various products is presented in Fig. 3. The highest inhibition rate of V_C_ was 95.75% at 30 μg/mL. The highest DPPH^⋅^ radical inhibition rates of DL and LT were 90.78% and 91.47% for ESE samples as well as 93.19% and 80.25% for WSE samples at 1000 μg/mL. The maximum inhibition rates of DR for ESE and WSE samples were only 70.94% and 69.24% at 1000 μg/mL. Therefore, we can draw the conclusion that DR presented much lower scavenging vigor than DL and LT for both water-soluble and ethanol-soluble extracts. At a certain concentration, the highest inhibition of DL and LT to free radicals was approximately equal to the level of V_C_. These active functions occurred in the leaf products of medicinal plants *A. membranaceus* and *C. pilosula*^20^*,* both of which were added to the list of Chinese medicine and food materials of the same origin.

The IC_50_ of ABTS and DPPH^⋅^ inhibition is shown in Table 3. In terms of the IC_50_ values of ABTS, those in DL and LT were higher than that of DR (P < 0.01). The maximum IC_50_ of WSE appeared in the DL sample (1101.69 μg/mL), but the maximum IC_50_ of ESE was noted in LT product (2163.60 μg/mL). The WSE of DR exhibited the highest IC_50_ values of DPPH^⋅^ than other two leaf products (P < 0.01), reaching a value of 40.97 μg/mL. Compared with IC_50_ of DR to DPPH^⋅^ (32.20 μg/mL), the values in ESE exhibited 64.41 μg/mL from LT and 53.90 μg/mL from DL, resulting in no significant difference from each other (P < 0.01), but the LT is significantly higher (*P* < 0.01). However, the IC_50_ to DPPH^⋅^ in ESE from the roots of *C. pilosula* was up to 417.91 μg/mL, and the IC_50_ was not detected within the experiment concentration extent^20^. From the above it could be deduced that the leaf of *A. membranaceus* have much more potential utilization value in developing antioxidant bio-products than the DR.

**Table 3:** Semi-lethal inhibitory concentration (IC_50_) of water and ethanol extracts of three products of *Astragalus membranaceus* (Fisch.) Bge. to ABTS and DPPH free radicals.

Extracts	Free radicals	IC_50_ of different products (μg/mL)		
		DL	LT	DR
WSE	ABTS	1101.69 ± 69.33^aA^	928.71 ± 110.74^aA^	511.98 ± 73.48^bA^
	DPPH	8.29 ± 0.81^cB^	17.25 ± 0.36^bB^	40.97 ± 3.63^aA^
ESE	ABTS	2113.98 ± 47.67^aA^	2163.60 ± 8.59^aA^	417.60 ± 78.67^bB^
	DPPH	53.90 ± 2.85^bA^	64.41 ± 2.30^aA^	32.20 ± 2.28^cB^

Data indicated mean ± SD. ABTS, 2,2-azino-bis-(3-ethylbenzothiazoline-6-sulfonic acid); DPPH, 2,2-diphenyl-1-picrylhydrazyl. Different superscript lowercase letters mean significant difference at P < 0.05, and superscript uppercase letters mean great significant difference at P < 0.01 in the same column, based on results of ANOVA-Duncan multiple comparisons by SPSS 20.0. The other abbreviation is the same with Table 1.

FRAP test was to determine whether the samples have efficient reducing power, thus further revealing their authentic antioxidant ability. The reducing power was calculated according to the absorbance of reaction mixture at 593 nm by V_C_ standard curve (*y* = − 0.013 + 0.006*x, r*^2^ = 0.998). The FRAP activity was expressed by *x* value calculated from above equation, finally shown in Table 4. When the concentration was at 20.0 mg/mL, we observed that the reducing powers of DL and LT to FRAP were very close both for WSE (546.66 μg/mL, 645.03 μg/mL) and for ESE (612.40 μg/mL, 645.33 μg/mL), and these trends also appeared the same to ABTS and DPPH^⋅^ inhibition. However, when the concentration of DR was at 20.0 mg/mL, the FRAP value for WSE and ESE were 51.33 μg/mL and 86.44 μg/mL, respectively. Obviously, DR showed much lower reducing power than DL and LT. The reducing powers of DL and LT were very similar, even approximated up to the level of V_C_ when getting at a certain concentration. These same trends of reducing power to FRAP also existed in products of *C. pilosula as observed* in a recent study^20^.

**Table 4:** Test results of ferric ion reducing antioxidant power (FRAP) tests for three products from *Astragalus membranaceus* (Fisch.) Bge. based on a V_C_ standard curve.

Products	FRAP tested result (μg/mL)	
	WSE	ESE
DL	546.66 ± 34.64^aA^	612.40 ± 28.73^aA^
LT	645.03 ± 46.95^aA^	645.33 ± 53.74^aA^
DR	51.33 ± 1.39^bB^	86.44 ± 1.75^bB^

The data are means ± SD. The superscript different small and capital letters in the same column indicate significant and great significant differences at P < 0.05 and P < 0.01, respectively, according to multiple comparison results of ANOVA-Duncan. The abbreviation items are the same with Table 1.

Regarding the scavenging activities of ABTS, DPPH^⋅^ and FRAP, the results indicate that DL and LT have similarly very high antioxidant activities, being able to reach the level of V_C_ at a certain concentration. DR antioxidant activities were significantly lower (P < 0.01)than the other two, and the inhibition capacities of ABTS, DPPH^⋅^ and FRAP were in accordance with the results of *Coronopus didymus*^31^. Therefore, DL and LT can be more widely and comprehensively developed and utilized in the future.

### Antimicrobial activity analysis

The extracts were diluted to gradient concentrations at 0.02–20.00 mg/mL based on 20.00 mg/mL concentration prepared by LT, DL and DR^20,27^. The results showed that the different concentrations of extracts from three (3) products had different inhibitory effects on six (6) strains. Table 5 showed the diameters of antibacterial zones determined in vitro experiment. The extracts of some samples showed inhibition to some strains at high concentration, but did not at lower concentration (Table 5). However, the extracts of other samples showed inhibition to some strains at low concentration, but did not at high concentration. DL and DR showed no antimicrobial activity both to *S. aureus* and *A. niger* but for *A. niger.* LT could play antibacterial activity to other bacteria and *yeast,* showing the varied antibacterial activities with the concentrations (Tables 5, 6).

**Table 5:** Zone diameters of antimicrobial inhibition to six testing microbial strains at different concentrations of three products from *Astragalus membranaceus* (Fisch.) Bge.

Products	Tested strains	Inhibition zone diameters (mm) of the tested concentrations (mg/mL) diluted from different samples										
		20.000	10.000	5.000	2.500	1.250	0.625	0.313	0.156	0.078	0.039	0.020
DL	Salmonella	7.78 ± 0.11	9.99 ± 0.13	9.14 ± 0.32	8.19 ± 0.14	9.48 ± 0.25	9.11 ± 0.30	–	–	–	–	–
	*Bacillus subtilis*	8.98 ± 0.02	8.52 ± 0.18	9.08 ± 0.07	8.36 ± 0.08	7.04 ± 0.19	7.96 ± 0.11	–	–	–	–	–
	*Staphylococcus aureus*	–	–	–	–	–	–	–	–	–	–	–
	*Escherichia coli*	–	–	–	–	–	8.15 ± 1.02	7.61 ± 0.23	8.07 ± 0.07	8.20 ± 0.76	7.82 ± 0.28	8.06 ± 0.87
	*Yeast*	–	–	–	–	–	8.55 ± 0.45	7.91 ± 0.15	7.27 ± 0.29	9.02 ± 0.34	8.62 ± 0.57	9.08 ± 0.32
	*Aspergillus niger*	–	–	–	–	–	–	–	–	–	–	–
LT	Salmonella	8.19 ± 0.08	8.23 ± 0.04	8.98 ± 0.14	7.42 ± 0.21	–	–	–	–	–	–	–
	*Bacillus subtilis*	8.74 ± 0.18	9.40 ± 0.36	8.18 ± 0.33	8.46 ± 0.12	8.52 ± 0.10	9.84 ± 0.01	–	–	–	–	–
	*Staphylococcus aureus*	–	7.19 ± 0.11	8.07 ± 0.17	8.17 ± 0.13	8.20 ± 0.19	7.34 ± 0.16	–	–	–	–	–
	*Escherichia coli*	–	–	–	–	–	–	7.55 ± 0.56	7.81 ± 0.06	8.01 ± 0.07	8.05 ± 0.13	7.62 ± 0.25
	*Yeast*	–	–	–	–	7.76 ± 0.29	8.18 ± 0.22	8.20 ± 0.36	8.66 ± 0.05	8.01 ± 0.18	8.93 ± 0.53	–
	*Aspergillus niger*	–	–	–	–	–	–	–	–	–	–	–
DR	Salmonella	7.77 ± 0.01	7.82 ± 0.18	6.83 ± 0.21	7.05 ± 0.23	–	–	–	–	–	–	–
	*Bacillus subtilis*	8.55 ± 0.18	8.88 ± 0.12	8.85 ± 0.09	7.64 ± 0.18	8.54 ± 0.35	8.66 ± 0.04	–	–	–	–	–
	*Staphylococcus aureus*	–	–	–	–	–	–	–	–	–	–	–
	*Escherichia coli*	–	–	–	–	–	–	8.01 ± 0.30	7.38 ± 0.20	8.13 ± 0.15	8.24 ± 0.40	8.55 ± 0.97
	*Yeast*	–	8.43 ± 0.69	7.45 ± 0.16	8.48 ± 0.82	7.44 ± 0.44	8.45 ± 0.36	8.90 ± 0.35	8.33 ± 0.21	7.62 ± 0.06	–	–
	*Aspergillus niger*	–	–	–	–	–	–	–	–	–	–	–

Each data indicates mean ± SD. “–” indicates no inhibition detected in the test. DL, dry leaves; LT, leaf tea; DR, dry roots.

**Table 6:** Maximum (Max) and minimum (Min) inhibition concentrations of three products from *Astragalus membranaceus* (Fisch.) Bge. to six tested microbial strains.

Products	Values (mg/mL)	*Salmonella*	*Bacillus subtilis*	*Staphylococcus aureus*	*Escherichia coli*	Yeast	*Aspergillus niger*
DL	Max	> 20.00	> 20.00	–	0.63	0.63	–
	Min	0.63	0.63	–	< 0.02	< 0.02	–
LT	Max	> 20.00	> 20.00	10.00	0.31	1.25	–
	Min	2.50	0.63	0.63	< 0.02	0.04	–
DR	Max	> 20.00	> 20.00	–	0.31	10.00	–
	Min	2.50	0.63	–	< 0.02	0.08	–

DL, dry leaves; LT, leaf tea; DR, dry roots. “–” indicates no inhibition detected in the test. “ < ” means less than a value, and “ > ” means more than a value.

The MAX and MIN of tested concentration extents are listed in Table 6. The extracts of DL, LT and DR all had antibacterial activities against Salmonella at a concentration of 20.00 mg/mL, however, their MIN antibacterial concentrations appeared at 0.63 mg/mL, 2.50 mg/mL, and 2.50 mg/mL, respectively, indicating DL has stronger bacteriostatic effect. The sensitivities of the extracts from DL, LT and DR to *B. subtilis* were quite similar, having 0.63 mg/mL for the MIN and at least 20.00 mg/mL for the MAX. All the extracts of DL, LT and DR have antibacterial effects on *E. coli* at low concentrations of 0.02 mg/mL. Differing from the MIN, the MAX concentration varied with the products. It was 0.63 mg/mL for DL, 0.31 mg/mL both for LT and DR products. The extracts of DL, LT and DR had antimicrobial effect on *yeast*, but the effects varied with the concentrations, and the MIN and MAX concentrations appeared at 0.02 mg/mL and 0.63 mg/mL for DL, 0.04 mg/mL and 1.25 mg/mL for LT, and 0.08 mg/mL and 10.00 mg/mL for DR. Among the extracts, only the extracts of LT showed antibacterial activity on *S. aureus*, and the effective concentrations ranged 0.63 mg/mL to 10.00 mg/mL. Therefore, DL and LT were more sensitive to some bacteria and *yeast*; this proved that they have stronger and efficient antibacterial activity (Table 6). On the basis of exploiting bioactive potential of wild radish, *Raphanus raphanistrum* using hydroethanolic and decoction extracts, Iyda found both the samples proved to inhibit several Gram-positive and Gram-negative bacteria and revealed antioxidant activity^21^, while cytotoxicity against non-tumour cell was not observed. In our study, a systematic toxicological test was carried out on the three products from medicinal plant *A. membranaceus* in vivo and the results reveal no toxicity.

## Conclusions

The usefulness of Astragali Radix root as valuable traditional Chinese medicine cannot not be underestimated. Its continuous root growth stimulation through the pruning of branches and leaves generates substantial amount of biomass. To promote the utilization of A. membranaceus leaves, the leaves were developed into a value added product—leaf tea. The evaluated chemical constituents, antioxidant capacities and antimicrobial activities of the leaves, leaf tea and roots were showed that the leaves of A. membranaceus had high nutritional value. Also a comparison of DL, LT and DR showed similarities in the chemical constituents of LT with DL. Compared with the DR, the nutrient contents in DL and LT exhibited higher bioactive chemicals (including polysaccharides, polyphenolic, flavonoids, protein and amino acids) and strong antioxidant effects. In this study, significantly higher protein values (DL: 28.21%, LT: 19.99%, DR: 14.52%), TPC (DL: 5.79%, LT: 5.26%, DR: 1.80%), TFC (DL: 9.68%, LT: 8.11%, DR: 1.90%) and total amino acids (DL: 24.18%, LT: 28.96%, DR: 8.89%) were detected in DL and LT. DL showed similar antioxidant and antibacterial activities as LT.

In addition TFC, POL, TPC, protein and amino acids, differing from the DL, LT exhibited stronger DPPH antioxidant activities and more broad inhibiting ability, an indication of its better health benefits.

Furthermore the two leaf products DL and LT are easy to obtain at certain concentration of antioxidant for best inhibition ability close to that of VC.

Compared with DL and DR, LT also has certain antimicrobial activities against bacteria and yeast.

It can be concluded that A. membranaceus leaves shows promising health benefits and can be effectively utilized as a medicinal plant as part of a varied healthy diet.
