# Author Correction: Antioxidant and antibacterial insights into the leaves, leaf tea and medicinal roots from *Astragalus membranaceus* (Fisch.) Bge.

**DOI:** 10.1038/s41598-021-00118-8

**Published:** 2021-10-12

**Authors:** Anim Okyere Samuel, Bao-Ting Huang, Yuan Chen, Feng-Xia Guo, Dou-Dou Yang, Jian-Qin Jin

**Affiliations:** 1 College of Life Science and Technology, College of Agronomy, Gansu Provincial Key Lab of Aridland Crop Science, Gansu Key Lab of Crop Genetic Improvement & Germplasm Enhancement, Gansu Provincial Key Lab of Good Agricultural Production for Traditional Chinese Medicines, Gansu Provincial Engineering Research Centre for Medical Plant Cultivation and Breeding, Gansu Agricultural University, Lanzhou, 730070 China; 2 CSIR-Oil Palm Research Institute, P.O BOX 74, Kusi, Ghana

Correction to: *Scientific Reports* 10.1038/s41598-021-97109-6, published online 04 October 2021

The original version of this Article contained an error in the Acknowledgments section.

“This work was supported by National Administration of Traditional Chinese Medicine (ZYBZH-Y-GS-11); Science and Technology Department of Gansu Province Support Project (1204FKCA123); Gansu Provincial Department of Agriculture and Rural Areas Foundation to Prof. Yuan Chen for a Chief Expert of Traditional Chinese Medicinal Industry in Modern Agricultural Industry System (GARS-ZYC-1) and once again to Prof. Yuan Chen for Special Advantage Agricultural Products in Gansu Province -Evaluation of Longxi Huangqi (TYNPZ2020-18), Gansu Zhongtian Pharmaceutical Co., Ltd on the GAP Base Construction of *Astragalus membranaceus* (2017-2020) and SRTP Project of Gansu Agricultural University (202111018, 202101045).”

now reads:

“This work was supported by Gansu Provincial Department of Agriculture and Rural Areas Foundation to Prof. Yuan Chen for Evaluation of Gansu Provincial Special Advantage Agricultural Product- Longxi Huangqi (TYNPZ2020-18) and Foundation for a Chief Expert of Traditional Chinese Medicinal Industry in Modern Agricultural Industry System (GARS-ZYC-1); National Administration of Traditional Chinese Medicine (ZYBZH-Y-GS-11); Science and Technology Department of Gansu Province Support Project (1204FKCA123); Gansu Zhongtian Pharmaceutical Co., Ltd on the GAP Base Construction of *Astragalus membranaceus* (2017-2020) and SRTP Project of Gansu Agricultural University (202111018, 202101045).”

The original Article has been corrected.

